# Supplementary material for: Perfect linear optics using silicon photonics
Source: Nat Commun. 2024 Jun 27;15:5468. doi: 10.1038/s41467-024-49768-y (PMC11211446; doi:10.1038/s41467-024-49768-y)
Supplement: Supplementary file 1 — Supplementary Information [file 41467_2024_49768_MOESM1_ESM.pdf]

Perfect Linear Optics using Silicon Photonics

This document provides supplementary material for the manuscript "**Perfect Linear Optics using Silicon Photonics**".

A. Passive characterization of the 4×4 crossbar and its elementary device

The initial conditions of the employed calibration model referred to as the "baseline" model were derived through the experimental characterization of a standalone 50 um SiGe electro-absorption modulator (EAM). Figure S1 illustrates the experimentally measured insertion loss (IL) and the achieved static extinction ratio (ER) values when the EAM was reverse biased at a voltage level that ranged between 0 and 3V in steps of 0.5V in the C-band transmission window.

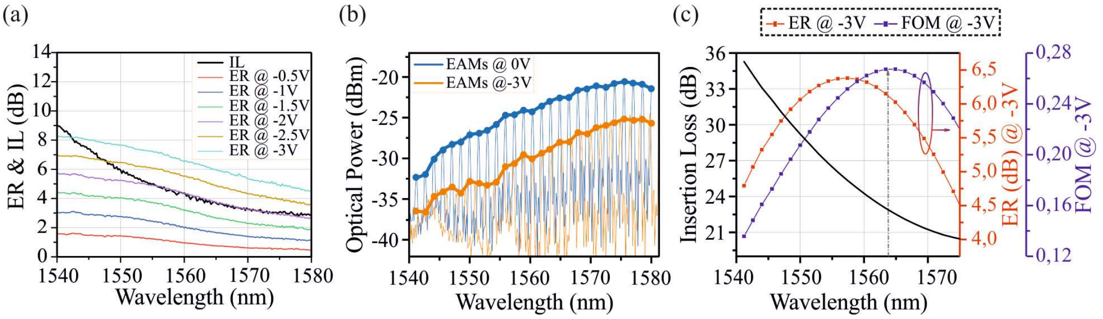

Fig. S1: (a) Standalone 50 um SiGe EAM characterization. Black solid line represents the insertion loss (dB), while colored solid lines correspond to the extinction ratio of the EAM at [-3, 0] V. (b) Optical spectrum of 4×4 Xbar's column #2 when constituent EAMs are driven at 0 and -3V. (c) Fitted IL, ER and FOM of column 2.

Followingly, the wavelength dependency of the 4x4 Xbar prototype , with the corresponding experimental setup illustrated in Figure S2, was evaluated through tuning all the node phase shifters to perform fully-constructive interference and measuring the resulting optical spectrum response across all 4 Xbar column outputs. Figure S1(b) indicatively illustrates the acquired spectra for Column #2's output, when all input vector EAMs were driven at 0V and all intra-column EAMs were biased at either 0V (blue curve) or 3V (orange curve). The observed resonant behavior can be avoided in future fabrication runs by matching the optical path lengths within the 4-branch multiport interferometer formed between the Xbar input and the column output. Fitting the acquired transfer functions envelope data to 2nd order polynomials, we calculate the IL and ER obtained for Xbar column#2 across the C-band. These metrics are plotted in Fig. S1(c) together with a figure-of-merit (FOM) that correlates ER and IL at the column output and is expressed as $\boldsymbol{FOM} = \boldsymbol{ER}(\boldsymbol{dB})/\boldsymbol{IL}(\boldsymbol{dB})$. A maximum FOM of ~0.26 is obtained at $\boldsymbol{\lambda} = \mathbf{1563}\ \boldsymbol{nm}$, providing the optimum operational wavelength.

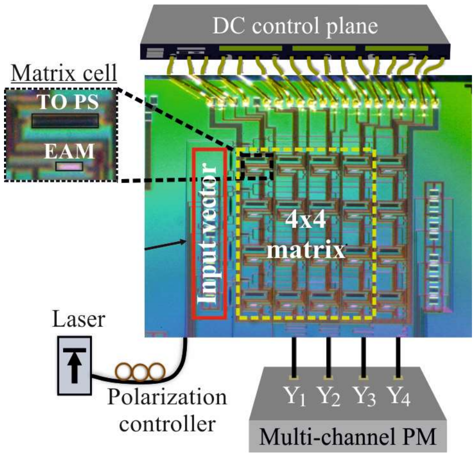

Fig. S2: Experimental setup for the characterization and programming of the 4×4 silicon photonic Crossbar. Inset: Elementary computing cell composed of a SiGe EAM and a Si TO PS.

B. Hardware aware programming model: Algorithm and computational complexity

[a]. Algorithm description

The first step of the 4×4 SiPho Xbar programming procedure towards assessing the EAMs' performance deviation from the standalone EAMs measurements and every hardware mis-uniformity, included the

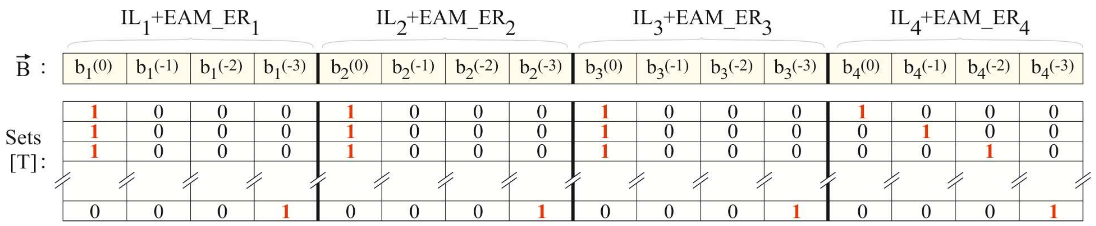

Fig. S3: Visual representation of the system of linear algebra matrices created to approximate the behavior of Xbar specifications (passive and active components).

experimental implementation of arbitrary linear transformations by biasing the #4 EAMs at each column with bias voltages in space $\boldsymbol{B} = \{\boldsymbol{0}, -\boldsymbol{1}, -\boldsymbol{2}, -\boldsymbol{3}\}\boldsymbol{V}$. This experiment provided us with #256 (=4^4) different programming sets and #16 unknown parameters for each individual column, defined by the IL of each row ($\boldsymbol{IL_r}$) and the attenuation induced by the respective EAMs when biased with a voltage $\boldsymbol{V} \in \boldsymbol{B}$. We express the unknown parameters in a 16-elements long vector of the form: $\vec{\boldsymbol{C}} = [\boldsymbol{b_1}(\boldsymbol{0}), \boldsymbol{b_1}(-\boldsymbol{1}), \boldsymbol{b_1}(-\boldsymbol{2}), \boldsymbol{b_1}(-\boldsymbol{3}), \boldsymbol{b_2}(\boldsymbol{0}), \cdots, \boldsymbol{b_4}(-\boldsymbol{3})]$, with $\boldsymbol{b_r}(\boldsymbol{V})$, corresponding to the $\boldsymbol{IL_r} + \boldsymbol{EAM\_ER_r}(\boldsymbol{V})$, of row #r $\in \{\boldsymbol{1},\boldsymbol{2},\boldsymbol{3},\boldsymbol{4}\}$ when the respective EAM is biased at V ∈ B volts. The electric field linear summation of each practical set (i.e., each set comprises a unique combination of bias voltages applied to the EAMs of different rows) of $\vec{\boldsymbol{C}}$ with the all-one input vector would yield the 256-elements long vector with $\vec{\boldsymbol{Y}} = \frac{\boldsymbol{1}}{\sqrt{\boldsymbol{4}}}[\boldsymbol{b_1}(\boldsymbol{0}) + \boldsymbol{b_2}(\boldsymbol{0}) + \boldsymbol{b_3}(\boldsymbol{0}) + \boldsymbol{b_4}(\boldsymbol{0}), \cdots, \boldsymbol{b_1}(-\boldsymbol{3}) + \boldsymbol{b_2}(-\boldsymbol{3}) + \boldsymbol{b_3}(-\boldsymbol{3}) + \boldsymbol{b_4}(-\boldsymbol{3})]$, which corresponds to the experimentally acquired values measured through our SiPho Xbar. Consequently, we create a 256:16 binary matrix *T* as depicted in Fig. S3, with the non-zero elements dictating which bias voltage was applied in which row's EAM. For instance, the second row of the matrix *T*, indicates that the EAMs of the first three rows are bias at 0Vs, while the EAM of the last row is biased at -1Vs. Using $\vec{\boldsymbol{C}}$, $\vec{\boldsymbol{Y}}$ and *T* we can, then, approach the overparametrized system of linear equations (16 unknowns with 256 equations) as a linear algebra problem with $\vec{\boldsymbol{Y}} = \boldsymbol{T} * \vec{\boldsymbol{C}}$ and exploit the least square method, to approximate the solutions $\vec{\boldsymbol{C}} = ((\boldsymbol{T^T} * \boldsymbol{T})^{-\boldsymbol{1}} * \boldsymbol{T^T}) * \vec{\boldsymbol{Y}}$.

HA-model algorithm

r : Crossbar row

c: Crossbar column

φ: Phase shift

w: Weight

EAM_rb: EAM reverse bias voltage (V)

```
1:  For r, c in [1, 4]:
        Tune φ(r, c).
2:  For r in [1, 4]:
3:      For c in [1, 4]:
4:          For EAM_rb(r, c) in [0, 3]:
5a:             Measure Y=Σ(c, EAM_rb(r, c))
5b:             Normalize to Σ to [0, 1]
6:  Solve Y=T*C to get w coefficients
7:  For c in [1, 4]:
8:      For r in [1, 4]:
9a:         Limit w(r, c, EAM_rb) to
            [min(w([1, 4], c, 0), max(w([1, 4], c, 3)]
9b:         Normalize to [0, 1]
10:         Find EAM_rb(r, c, w)
```

Fig. S4: HA-model algorithmic description

Figure S4 illustrates a generalized form of the algorithmic procedure followed during the application of the HA-model, with the brackets corresponding to the algorithmic description:

1. [1]. The Phase Shifters (PS) of each Crossbar column are fine tuned so that nested MZIs interfere constructively.
2. [2-5b] Pilot linear transformation measurements are performed, by permutating the voltages of each column's constituent EAM-based nodes. The end results are normalized to [0,1].
3. [6] The least square method is employed to calculate the weight contribution of each row node as a function of the applied reverse voltage.
4. [9a-9b] Limit each column's constituent EAM operating voltages to [minimum achievable transmission of all row contributions at -3V, maximum achievable transmission of all row contributions at 0V] and normalize the vectors to [0,1]
5. [10] (i) Find the row contribution vs EAM bias voltage fit and calculate the inverse transfer functions to transform voltage values to weighing factors and find each EAM's voltage range (ii) Normalize transmission on new EAMs bias voltage

ranges and "fine-fit" the rows' contribution vs EAM bias voltage relation. (iii) Normalize the rows contribution values to [0, 1] and calculate the inverse transfer function. The latter corresponds to the required look-up table between the weigh values and the EAM-voltages.

The related results for all four Xbar's columns, after the application of the square method and the final look up tables are illustrated in Fig. S5 (a) and (b) respectively.

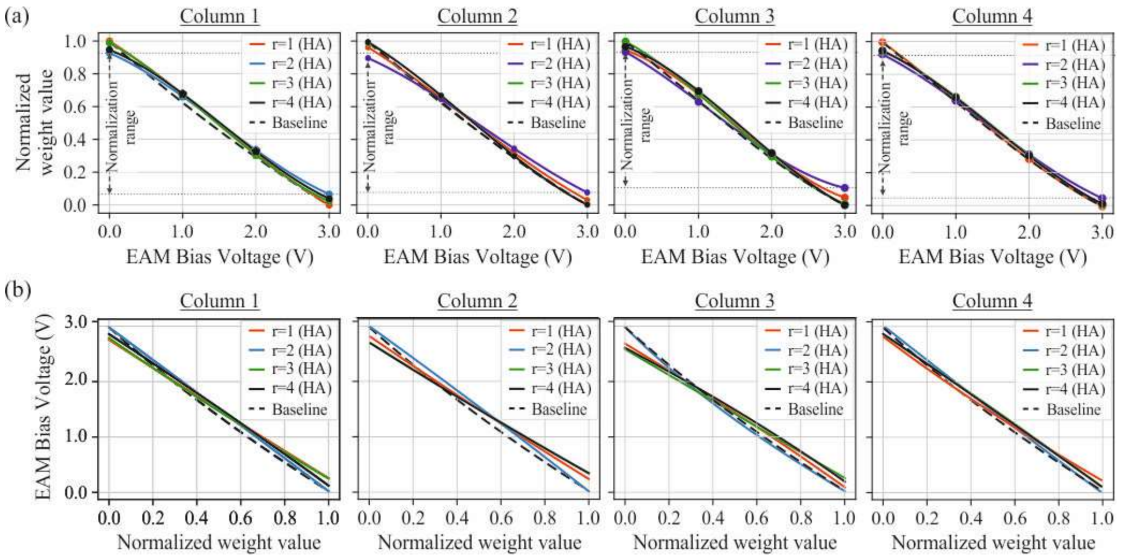

Fig. S5: (a) Experimentally derived relation of row's weighting values versus EAMs bias voltage for all 4 Xbar columns. (f) EAM bias voltage vs weight value "look-up" table for all 4 Xbar columns

[b]. Computational complexity

In order to clarify our approach, we begin by describing the difference between the programming and calibration computing complexity of a photonic matrix and report the respective metrics. The programming complexity is associated with the procedure required to imprint an arbitrary transformation matrix into the photonic hardware. In the prominent SVD-based approach, transferring a N×N transformation matrix into the photonic accelerator, necessitates the calculation of the constituent matrices of the SVD decomposition scheme, a mathematical operation typically having a programming complexity of $O(N^3)$ [3]-[4]. In the case of our proposed photonic Xbar architecture, its bijective nature implies that the programming complexity is reduced to a memory look-up operation, as each weight node has a unique relationship with the targeted weight matrix coefficients. This operation for a single node has a computational complexity of O(1) [5], a complexity that can be maintained for a N×N matrix when assuming a parallel computational system or reach O(N) when assuming a column parallel memory look-up system.

On the other hand, the calibration complexity of a photonic matrix is related to the algorithm that approximates and assimilates in the programming procedure the inevitable fabrication-induced photonic device variations. The related complexity for SVD-based approaches has been extensively studied, with an interesting comparative study presented in Appendix B of [6]. In the case of our HA-model, the calibration complexity is mainly dictated by the computational complexity of the least-squares method applied to calculate the vector $\vec{\boldsymbol{C}}$, that expresses the transmission coefficients of each constituent node. Generalizing the procedure described in the B part of the Supplementary material for an N×N Xbar array and $\boldsymbol{k}$ possible values for the EAM voltage space, such as $B = \{0_1, \cdots, -3_k\}\ V$, we can conclude to the following linear system:

$$\vec{\boldsymbol{Y}} = \boldsymbol{T} * \vec{\boldsymbol{C}} \quad (1)$$

,with $\vec{\boldsymbol{Y}}$ representing the output vector with a dimensionality of $[k^N \times 1]$ and k^N related to the possible combinations of voltages applied in the each Xbar column constituent EAM nodes, reaching up to -3V reverse bias voltages for safety reasons, $\boldsymbol{T}$ corresponding to the binary-based expression of the node contribution with a dimensionality of $[k^N \times k \cdot N]$ and the unknown vector $\vec{\boldsymbol{C}}$ expressing the $\boldsymbol{k}$ coefficients for each constituent node with dimension of $[k^N \times 1]$. Expressing equation 1 including the related dimensions leads to:

$$\vec{\boldsymbol{Y}}[k^N \times 1] = \boldsymbol{T}\,[k^N \times k \cdot N] * \vec{\boldsymbol{C}}[k \cdot N \times 1] \tag{2}$$

with the computational complexity of solving equation (2) using the least-square method mainly dictated by the dimensionality of the $\boldsymbol{T}$ matrix and typically approximated with a complexity of $O(N^3)$ for an M×N matrix [7]. As such, the generalized form of the computation complexity of the HA-model is $O(k \cdot N)^3$. From this expression we can deduce that the main contributors to the computational complexity are: (i) The value $\boldsymbol{k}$ represents the number of values describing the EAM-based node transfer function (ii) The row dimension of the matrix $\boldsymbol{T}$**,** dictated by both the $\boldsymbol{k}$ value and also the required number of equations for approximating $\vec{\boldsymbol{C}}$ with high precision.

Considering the required number of $\boldsymbol{k}$ values to accurately approximate the transfer function of the EAM-based node, we plot in Fig. S6 (a) the normalized transfer function of a stand-alone 50 um long EAM for three different wavelengths, located in the periphery of its maximum FOM operating point. The experimentally derived transfer function comprised 12 points equally spaced in the range R =[0,3] V, with R representing the applied reverse bias voltage. Figure S6 (b) illustrates the mean absolute error achieved when fitting an increasing number of equally space data points using a 2nd order polynomial and comparing with the experimentally derived performance. As we can observe, even with $\boldsymbol{k \leq 4}$ the related improvement in precision is less than 0.3% for all 3 wavelengths, showcasing that even 4 data points are sufficient to accurately model an EAM-based node.

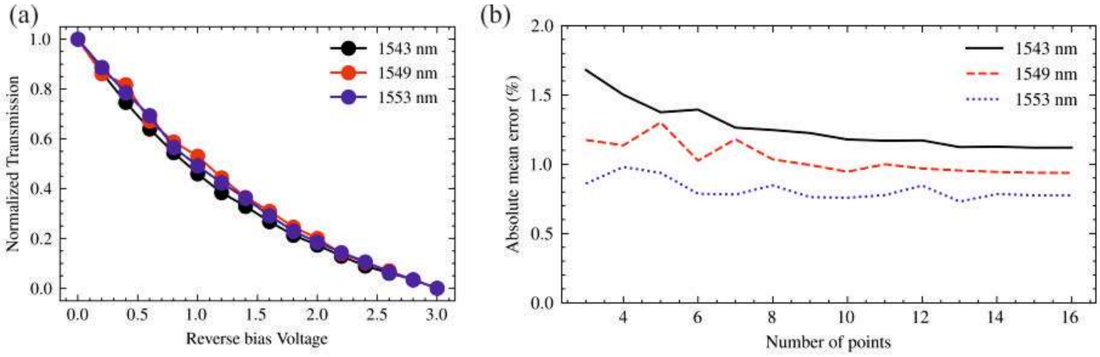

Fig. S6: (a) Normalized 50-um EAM transfer function for three different wavelengths (b) Absolute mean error versus number of points used for fitting a 2nd order polynomial to the experimental data.

Finally, towards quantifying the programming complexity and the convergence credentials of the employed HA calibration model when reducing the row dimensions of matrix **T**, we performed a simulation analysis where we trained the HA model using only part of the calibrating dataset, yet always keeping the length of the utilized dataset greater than the number of the unknown parameters (>16). The absolute programming error of the model was then recorded. More specifically, we split the calibration dataset in two parts with the 80% being utilized for calibrating the model and the residual 20% for validation. The dataset employed for calibration purposes was formed by firstly selecting 16 linear transformations, where all the unknown parameters are contributing and, subsequently, adding random samples of the remaining batch. At each step, we record the absolute programming error of each Xbar column. The simulation results are depicted in Fig. S7. The errors calculated at columns 1-4 are illustrated with the blue, orange, red and green bold solid curves, respectively, while their minimum error is highlighted by the respective horizontal light solid lines. As observed, in the best case the minimum absolute programming error can be achieved using less than 5%, on top of the necessary #16 samples from the training dataset, with the worst case requiring ~63% extra samples. This can be translated to a minimum required dataset comprising 10-65% of the 256 experimentally obtained samples. Setting an error margin of 10% relative to the minimum achieved error, reveals that even when

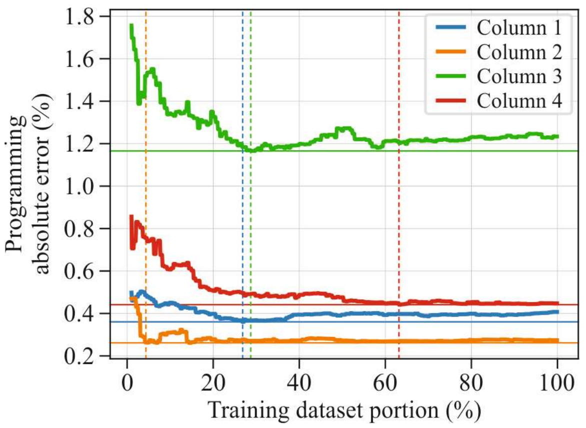

Fig. S7: Quantitative illustration of calibration model's dataset length requirement towards achieving the minimum absolute programming error at each Xbar column. X-axis: Percentage of the #240 linear transformations dataset capitalized to train Xbar's calibration model.

using <17% of the acquired 256 measurements, high converge and low absolute error can still be achieved.

[c]. Validation across multiple chips

In order to assess the effectiveness of our HA-programming model in a larger number of samples, two additional 4×4 Xbar chips were measured and calibrated. Figure S8 (a)-(c) puts in juxtaposition the experimentally obtained absolute error when programming the Xbars using the baseline and HA model for all the columns. The reported absolute mean error and std values for all the 4 columns of the 3 different chips for the HA-model were measured to be μ=[0.74,0.81,1.1] and σ=[0.48,0.53,0.8]%, showcasing a small divergence range and validating the capabilities of the HA model in minimizing the respective programming error. Figure S8 (d) illustrates a histogram of the experimentally acquired fidelity values for 10.000 arbitrary matrix transformation for all 3 Xbar chips. The 3 different chips achieved matrix fidelities of 99.997±0.002%, 99.995±0.003%, 99.992±0.005% respectively, validating the potential of our HA-model and fidelity restoration mechanism in achieving almost unity fidelity values with small variance across different samples.

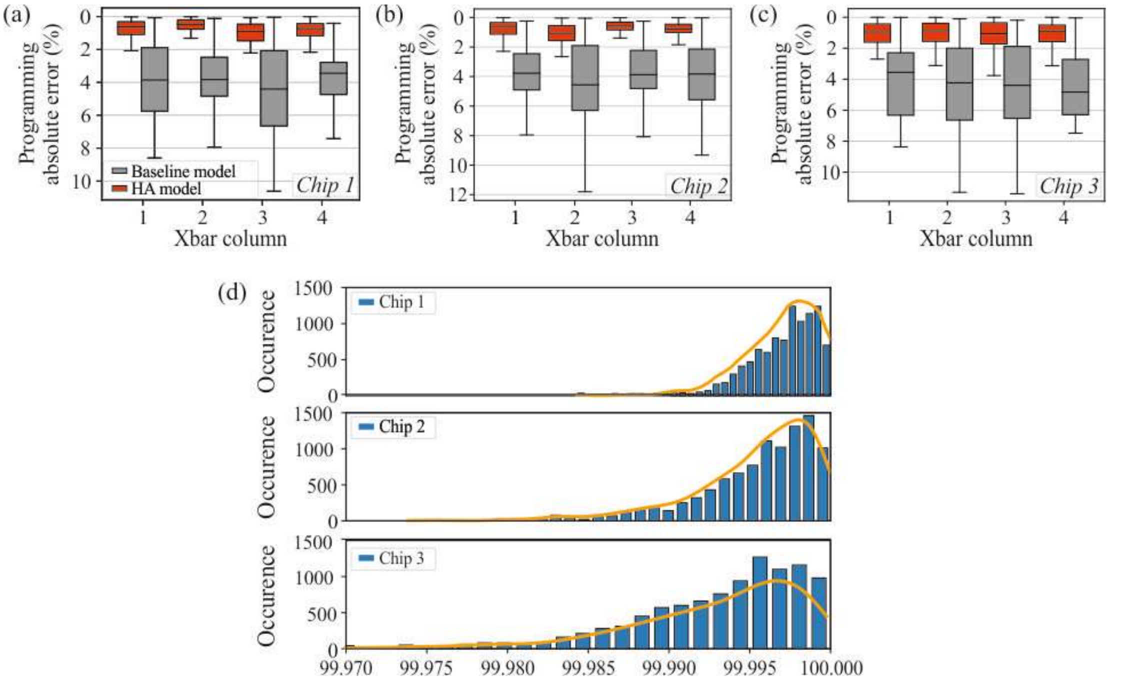

Fig. S8: (a) - (c) Experimentally obtained absolute error when programming the Xbar by the baseline and HA model for all columns and 3 different chips. (d) Experimentally obtained fidelity in the execution of #10000 arbitrary linear transformation matrices for 3 different chips, after employing the HA-programming model and fidelity restoration.

C. Power consumption analysis

The deployment of SiGe EAMs for the encoding of the input vector and transformation matrix elements, apart from the high bandwidth of >50 GHz, equips the Xbar architecture with ultra-high energy efficient computing nodes. The static power consumption of the EAMs can be calculated through $\boldsymbol{EAM_{static}} = \left(\frac{1}{2}\right) * \boldsymbol{P_{in,mW}} * \boldsymbol{R} * \boldsymbol{V_{static}} = \mathbf{24}\ \boldsymbol{\mu W}$ [1], with $\boldsymbol{P_{in,mW}}$ corresponding to the incident power at the EAM input port equal to ~3 mW, R the responsivity of the device of $\boldsymbol{R} = \mathbf{0.8}\ \boldsymbol{A/W}$, and $\boldsymbol{V_{static}}$ the average driving voltage, equal to 1.5 V when considering a uniform distribution in the range B[0-3]. This translates into a Xbar transformation matrix power consumption of only <0.4 mW.

Operating the EAM nodes dynamically, would unlock several capabilities [2] and applications where the Xbar architecture can be employed. Assuming a driving voltage of 1 Vpp, each EAM operating at 50 GHz would consume $\boldsymbol{EAM_{dynamic}} = \boldsymbol{CR} * \left(\frac{1}{4}\right) * \boldsymbol{C} * \boldsymbol{V_{dynamic}^2} = \mathbf{0.25}\ \boldsymbol{mW}$ on top of its static power consumption, concluding to an overall average Xbar transformation matrix power consumption of ~4.4 mW. It is worth mentioning that in the case of unbalanced arms between the nested MZIs' arms, the power consumption of the Xbar transformation matrix programming should, also, include the energy consumed to tune the silicon TO PSs, requiring 4 mWs for a full π-shift each.

References

1. M. Pantouvaki, et. al., “Active Components for 50 Gb/s NRZ-OOK Optical Interconnects in a Silicon Photonics Platform.” Journal of Lightwave Technology, vol. 35, no. 4, pp. 631-638 (2017).
2. G. Giamougiannis et al., "Universal Linear Optics Revisited: New Perspectives for Neuromorphic Computing With Silicon Photonics," in IEEE Journal of Selected Topics in Quantum Electronics, vol. 29, no. 2: Optical Computing, pp. 1-16, March-April 2023
3. G. H. Golub and C. F. Van Loan, "Matrix Computations," 4th ed. Baltimore, MD, USA: Johns Hopkins University Press, 2013
4. S. Pai et al., "Parallel Programming of an Arbitrary Feedforward Photonic Network," in IEEE Journal of Selected Topics in Quantum Electronics, vol. 26, no. 5, pp. 1-13, Sept.-Oct. 2020
5. G. Giamougiannis, A. Tsakyridis, Y. Ma, A. Totović, M. Moralis-Pegios, D. Lazovsky, et al., A coherent photonic crossbar for scalable 301 universal linear optics, Journal of LightwaveTechnology. 41 (2023) 2425–2442. doi:10.1109/jlt.2023.3234689.
6. R. Hamerly, S. Bandyopadhyay, and D. Englund, “Stability of self-configuring large multiport interferometers,” Physical Review Applied, vol. 18, no. 2, 2022
7. G. H. Golub and C. F. Van Loan, Matrix Computations, 4th ed. Baltimore, MD, USA: Johns Hopkins University Press, 2013
